# Supplementary material for: Diversity of Late Blight Resistance Genes in the VIR Potato Collection
Source: Plants (Basel). 2023 Jan 6;12(2):273. doi: 10.3390/plants12020273 (PMC9862067; doi:10.3390/plants12020273)
Supplement: Supplementary file 1 [file plants-12-00273-s001.zip › plants-2120519-supplementary.pdf]

Table S1. *Solanum* species and accessions studied

Series	Species	Plant ID	Accessions No in VIR catalogue	Number of tests	
				Late blight resistance	SCAR markers of the *Rpi* genes
Bulbocastana	*S. bulbocastanum*	GRA 300B	k-24862, k-21278	0	2
		HAW 1581	k-24863, k-25351	4	1
		HAW 1586	k-23174	2	1
		HAW 1588	k-24864	0	2
		HAW 1589	k-24865	0	1
		HAW 1591	k-24866, k-21274	4	8
		HAW 1593	k-24367, k-21276, k-23178	0	1
		HAW 1595	k-24867, k-23180	0	1
		HAW 1719	k-21266	1	1
		HAW 1796	k-24868, k-23181	0	6
		OCH 14142	k-19981	1	1
		ROC S-360 × S-395	k-24854, k-23165	0	1
		ROC S-361 × S-359	k-24855, k-25350	2	2
		ROC S-362×S-361	k-24856, k-23167	0	1
		ROC S-395 × S-398	k-24858	0	1
		ROC S-395×S-359	k-24857	0	1
		ROC S-397× S-359	k-24859	0	1
		ROC S-397× S-360	k-24860	0	2
		ROC S-398 × S-359	k-24861	0	1
		**Total 19**		**14**	**35**
Pinnatisecta	*S. pinnatisectum*	HAW 1455	k-24949	0	1
		HAW 1456	k-24950	0	1
		HAW 1505	k-24415	1	1
		HAW 1665	k-23569	1	1
		nd	k-24239	1	1
		nd	k-24243	1	1
		Tarn 205a	k-19328, k-21955	2	2
		WRF 343	k-24953	0	1
		**Total 8**		**6**	**9**
	S. cardiophyllum	GRA s.n.	k-16828	1	2
		GRA s.n.	k-24375	1	1
		GLKS 6/23	k-23030	1	1
		GLKS 95	k-24203	1	1
		GLKS 109	k-24206	1	1
		nd	k-4464	0	1
		**Total 6**		**5**	**7**
	S. cardiophyllum subsp. ehrenbergii	GRA 371	k-23279, k-23270	1	1
		HAW 1100	k-23276	0	1
		HAW 1421	k-18085, k-19061, k- -19257	1	4
		HAW 1427	k-18224	1	1
		HAW 1428	k-18225, k-24373	2	2
		HAW 1440	k-23277	2	2
		HAW 1443	k-24572	2	2
		HAW 1458	k-21301	1	1
		TARN 241D	k-22684	0	1

		WRF 1277	k-18086	1	1
		GLKS 2152	k-24207	1	1
		**Total 11**		**12**	**17**
	S. jamesii	BKPF 080	нет	0	1
		GRA 388 × 381	k-15203, k-23397, k-24920	5	4
		GRA 388×384	k-23398	1	1
		nd	k-22619	0	1
		SBV 21	k-24397	0	1
		SD J 116	k-24921	0	1
		SD S 91 x W 62	k-24922	1	1
		SD V 63 x S8	k-24923	1	1
		UGR 7-78	k-23399	1	1
		**Total 9**		**9**	**12**
	S. stenophyllidium	GRA 348×345	k-20105	1	1
		HAW 1234	k-24962	0	1
		nd	k-24255	1	1
		**Total 3**		**2**	**3**
Yungasensa	*S. chacoense*	CHC 2430/3	k-2861	2	2
		CPC 1317.1	nd	0	1
		HHR 3700	k-7394	2	0
		nd	k-2732	0	2
		nd	k-2739	0	1
		nd	k-3060	0	2
		OKA 6116	nd	0	1
		OKA 6888	k-19069, k-19265, k-21324	0	1
		WRF 362	k-21308	2	1
		**Total 9**		**6**	**11**
Maglia	*S. maglia*	Cor C.1	k-24604	0	1
		nd	k-24601	0	1
		CPC 2057	k-2883	0	1
		**Total 3**		**0**	**3**
Tuberosa (wild species)	*S. alandiae*	HHA 6526a	k-21240	1	2
		HHA 6662	k-20408	1	1
		OCH 12014	k-18473, k-19443	0	2
		**Total 3**		**2**	**5**
	S. avilesii	HHA6519	k-20884	1	1
		HHA 6521	k-20158	1	1
		**Total 2**		**2**	**2**
	S. berthaultii	EBS 1799	k-23146	0	3
		EBS 1846	k-21247, k-24267	2	2
		GKLS 7219	k-23047	6	1
		HHCH 4531C	k-19961	2	2
		OCH s.n.	k-23150	2	1
		UGN 4560 A	k-19243	0	1
		UGN 4561	k-24270	2	1
		**Total 7**		**8**	**11**
	S. microdontum	HHR 3690	k-18126	0	1
		HOHH 6012	k-25390	2	1
		OCHS 15534	nd	0	1
		nd	k-16799	0	1
		nd	k-23055	0	1
		nd	k-25385	2	2

		CIP761088	-	0	1
		CIP 762314	-	0	1
		**Total 8**		**4**	**9**
	S. microdontum subsp gigantofillum, simplicifolium syn.	GLk-S 62.31.7.1	k-5399	2	1
		GLKS 62.31.19.3	k-5414	1	1
		WRF 151	k-21499	1	1
		OKA 5614	k-21504	1	1
		**Total 4**		**5**	**4**
	S. sucrense	nd	k-9753	0	1
		nd	k-9247	0	1
		**Total 2**		**0**	**2**
	S. venturii	HHR 3741	k-25398	3	1
		HOHH 6033	k-25396	0	1
		OKA 4388×4392	k-12658	1	1
		OKA 4388×4404	k-25394	7	1
		OKA 7584×7585	k-25397	6	2
		**Total 5**		**17**	**6**
	S. vernei	EBS 181	k-23767	1	1
		OKA 4477	k-11438	2	1
		OKA 5700	k-23771	0	1
		OKA 7502	k-20332	2	1
		HAW 3505	k-18159	0	1
		**Total 5**		**5**	**5**
	S. verrucosum	CPC 1340.2	k-24991	2	2
		COR 14252	k-21695, k-24990	1	4
		GRA 327	k-24992	0	1
		HAW 1528	k-24993	0	1
		HAW 1658	k-24995	1	2
		SHGRF 4019	k-23015	1	1
		TRN 91 F	k-24313	1	1
		UGN 1289	k-23760, k-24996	1	2
		nd	k-24315	1	1
		**Total 9**		**8**	**15**
Tuberosa (cultivated species)	*S. phureja*	DOP 130 × 131	-	0	1
		OCH 5156	k-16533	0	1
		CIP 705164	-	0	1
		nd	k-6502	2	1
		nd	k-8940	5	1
		nd	k-17618	2	1
		**Total 6**		**9**	**6**
	S. stenotomum	nd	k-9278	2	1
		nd	k-17486	2	1
		**Total 2**		**4**	**2**
Demissa	*S. demissum*	BB s.n.	k-23315	1	1
		COR 14283	k-23306, k-24890	0	2
		COR 14266	k-21365	0	1
		COR 14200	k-24378, k-24885	0	1
		COR 14240	k-24887	4	3

		COR 14378	k-24891	0	1
		COR 14199	k-24884	0	1
		COR 14244	k-21364, k-24888	1	1
		COR 14432	nd	0	1
		COR 14379	nd	0	1
		CPC 7.3	k-24894	0	2
		CPC 21.5	k-21371, k-24895	1	2
		EBS 14/144	k-23311	0	1
		EBS 17/28	k-21367, k-24892	0	1
		EBS 46/33	k-24903	0	1
		HAW 1079	k-24893	0	1
		HAW 1108	nd	0	1
		HAW 1295	k-15173	0	1
		HAW 1296	k-15174	2	1
		HAW 1601	k-15175, k-24898	1	2
		HAW 1657	k-24899	0	1
		OCH 14156	k-18487	1	2
		OCH 14217	k-18521	1	1
		ROC S-38	k-24897	0	1
		ROC S-74	k-23316	0	1
		RDD 886	k-23320	0	1
		SHGRF 4010	k-24901	0	1
		SHGRF 4064	k-24902	0	1
		SHGRF 4261	nd	0	1
		TRHRG 123	k-19997	0	1
		TRHRG 150	k-19998	0	1
		**Total 31**		**12**	**38**
Longipedicellata	*S. stoloniferum*	CB 82-502	k-21618	0	1
		CCC 605B	k-24969	0	1
		COR 14246	k-18925	0	1
		COR 14247	k-24964	0	1
		COR 14263	k-23648	0	1
		COR 14270	k-21616	1	1
		COR 14271	k-15296	0	1
		CPC 1333.2	k-24968	0	1
		CPC 2093	k-24967	0	1
		CPC 9	k-23652	2	2
		EBS 2626	nd	0	1
		EBS 2942	nd	0	1
		GRA 222	k-24972	2	2
		GRA 334	k-23662	0	1
		GRA 341	k-24973	0	1
		GRA 366	k-19196, k-24420	3	4
		HAW 1107	k-24966	1	1
		HAW 1209	k-24975	0	1
		HAW 1392	nd	0	1
		HAW 1452	k-24977	0	1
		HAW 1520	k-20944, k-24976	4	4
		nd	k-3533	0	1
		nd	k-3336	0	1
		nd	k-3360	3	1
		nd	k-3554	0	1
		nd	k-24263	2	2

		ROC S-37	21619	0	1
		ROW 7	24980	0	1
		RSSV 926	nd	0	1
		SHGRF 4278	nd	0	1
		TRHRG 110	nd	0	1
		TRHRG 134	nd	0	1
		TRHRG 21	k-20106	1	2
		TRHRG 298	nd	0	1
		TRHRG 86	nd	0	1
		TRN 187	k-24981	1	2
		UGN 1288	k-24978	0	1
		**Total 37**		**20**	**48**
	S. polytrichon	GRA 278	k-24298	1	1
		GRA 315	k-16905	1	1
		GRA 356	k-23556	1	1
		HAW 1090	k-24410	1	1
		HAW 1467	k-19164	0	1
		HAW 1468	k-23561	1	1
		HAW 1669	k-18142	1	1
		nd	k-24462	1	2
		nd	k-24463	3	2
		OCH 14178	k-23563	0	1
		ROW 6	k-19333	0	1
		W78	k-8815	3	1
		**Total 12**	-	**13**	**14**
		**Total**			
**7**	**20**	**201**	-	**163**	**264**

Table S2. Screening *S. bulbocastanum* with SCAR markers of the *Rpi* genes and for late blight resistance

Plant ID	Accessions No in VIR catalogue (No of genotype)	LB resistance *	*Rpi-R1*	*Rpi-R2/Rpi-blb3*		*Rpi-R3a*	*Rpi-R3b*	*Rpi-R8*	*Rpi-blb1/Rpi-sto1*		*Rpi-blb 2*	*Rpi-vnt1*
			SCAR markers of the *Rpi* genes									
			Rpi-R1-1205	Rpi-R2-1137	Rpi-blb3-305	Rpi-R3a-1380	Rpi-R3b-378	Rpi-R8-1258	Rpi-blb1-821	Rpi-sto1-890	Rpi-blb2-976	Rpi-vnt1-612
ROC S-361 × S-359	24855, 25350, two genotypes	6-7	0	1	1	0/1	1	0	1	1	1	1
ROC S-362×S-361	24856	n.d.	0	0	1	1	1	0	0	0	1	1
GRA 300B	24862	n.d.	0	1	1	0	1	0	1	1	0	1
HAW 1591	24866 (298)	n.d.	0	0	0	0	0	0	1	1	1	1
"	24866 (329)	n.d.	0	0	1	0	0	0	1	1	1	1
"	24866 (D21-240)	4	0	1	1	1	1	0	0	0	1	1
HAW 1595	24867	n.d.	0	1	0	1	0	0	1	1	0	1
HAW 1588	24864 (S-3)	n.d.	0	1	1	0	0	0	1	1	1	1
"	24864 (11)	n.d.	0	0	0	0	1	0	0	0	1	1
ROC S-397× S-360	24860	n.d.	0	0	1	1	1	0	1	1	1	1
HAW 1796	24868 (330)	n.d.	0	0	1	0	0	0	0	0	1	1
"	24868 (331)	n.d.	0	0	1	0	0	0	0	0	1	1
ROC S-397× S-359	24859	n.d.	0	0	1	0	0	0	0	0	0	1

* Points by the 1-9 scale (1, susceptible; 9, resistant), nd – no data

Table S3. Screening *Solanum* species *Pinnatisecta* series with SCAR markers of the *Rpi* genes and for late blight resistance

Species	Plant ID	Accessions No in VIR catalogue	LB resistance	***Rpi-R1***	***Rpi-R2/Rpi-blb3***		***Rpi-R3a***	***Rpi-R3b***	***Rpi-R8***	***Rpi-blb1/Rpi-sto1***		***Rpi-blb2***	***Rpi-vnt1***
				SCAR markers of the *Rpi* genes									
				Rpi-R1-1205	Rpi-R2-1137	Rpi-blb3-305	Rpi-R3a-1380	Rpi-R3b-378	Rpi-R8-1258	Rpi-blb1-821	Rpi-sto1-890	Rpi-blb2- 976	Rpi-vnt1-612
S. pinnatisectum	Tarn 205a	19328	4	0	0	0	0	0	0	0	0	0	1
"	"	21955	5	0	1	0	0	0	0	0	0	0	1
"	HAW 1665	23569	5	0	0	0	0	0	0	0	0	0	1
"	nd	24239	7	0	1	0	0	0	0	0	0	0	1
"	nd	24243	8	0	0	0	0	1	0	0	0	0	1
"	HAW 1505	24415	7	0	1	1	0	1	0	0	0	0	1
S. cardiophyllum	nd	4464	nd	0	0	1	0	1	0	0	0	0	1
"	GRA s.n.	16828	7	0	0	0	0	0/1	0	0	0	0	1
"	GRA s.n.	24375	8	0	0	1	1	0/1	0	0	0	0 / 1	1
"	nd	23030	3	0	1	0	0	0/1	0	0	0	0	0
"	nd	24203	5	0	0	0	0	0/1	0	1	1	0	1
"	nd	24206	9	0	0	0	0	0/1	0	0	0	0	0
S. cardiophyllum subsp. ehrenbergii	GRA 371	23279	4	0	1	0	0	0	0	0	0	0	1
"	HAW 1100	23276	nd	0	1	0	0	0/1	0	0	0	0	1
	HAW 1427	18224	8	0	0/1	0	0	0	nd	0	0	0	nd
"	HAW 1428	18225	5	0	1	1	0	0/1	0	0	0	0	1
"	WRF 1277	18086	5	0	0	0	1/0	0/1	0	0	0	0	1

Table S4. Screening *S. chacoesne* (*Yungasensa* series) with SCAR markers of the *Rpi* genes and for late blight resistance

Plant ID	Accessions No in VIR catalogue (No of genotype)	LB resistance	***Rpi-R1***	***Rpi-R2/Rpi-blb3***		***Rpi-R3a***	***Rpi-R3b***	***Rpi-R8***	***Rpi-blb1/Rpi-sto1***		***Rpi-blb2***	***Rpi-vnt1***
			SCAR markers of the *Rpi* genes									
			Rpi-R1-1205	Rpi-R2-1137	Rpi-blb3-305	Rpi-R3a-1380	Rpi-R3b-378	Rpi-R8-1258	Rpi-blb1-821	Rpi-sto1-890	Rpi-blb2-976	Rpi-vnt1-612
OKA 6888 BAL 78155	19069, 19265 and 21324, three genotypes	nd	0	0	0	0	1	0	0	0	0	0
CPC 1317.1	no	"	0	0	0	0	1	0	0	0	0	0
OKA 6116	no	"	0	0	0	0	1	0	0	0	0	0
nd	2732 (537-4)	"	0	0	0	0	0	0	0	0	0	0
"	2732 (537-13c)	"	0	0	0	0	0	0	0	0	0	0
nd	3060 (538-8)	"	0	0	0	0	0	0	0	0	0	0
"	3060 (538-11)	"	0	0	0	0	0	0	0	0	0	0
CHC 2430/3	2861 (539-9)	2.3	0	0	0	0	0	0	0	0	0	0
"	2861 (539-12)	2.5	0	0	0	0	1	0	0	0	0	0
nd	2739 (C6)	nd	0	0	0	0	0	0	0	0	0	0
WRF - 362	21308	5.0	0	0	0	0	0	0	0	0	0	0

Table S5. Screening *Solanum* species *Tuberosa* (wild species) series with SCAR markers of the *Rpi* genes and for late blight resistance

Species	Plant ID	Accessions No in VIR catalogue (No of genotype)	LB resistance	***Rpi-R1***	***Rpi-R2/Rpi-blb3***		***Rpi-R3a***	***Rpi-R3b***	***Rpi-R8***	***Rpi-blb1/Rpi-sto1***		***Rpi-blb2***	***Rpi-vnt1***
				SCAR markers of the *Rpi* genes									
				Rpi-R1-1205	Rpi-R2-1137	Rpi-blb3-305	Rpi-R3a-1380	Rpi-R3b-378	Rpi-R8-1258	Rpi-blb1-821	Rpi-sto1-890	Rpi-blb2-976	Rpi-vnt1-612
S. alandiae	OCH 12014	18473, 19443	n.d.	0	0	0	0	0	0/1	0	0	0/1	0/1
"	HHA 6526a	21240	5	0	0	0	0	0	0	0	0	0	0
"	HHA 6662	20408	4	0	0	0	0	0	1	0	0	0/1	0
S. avilesii	HHA 6519	20884	4	0	1	0/1	0	0	1	0	0	1	1
"	HHA 6521	20158	7	0	0	0	0	0	1	0	0	1	1
S. berthaultii	HHCH 4531C	19961	7- 9	0	0	0	0	0	nd	0	0	0	0
"	"	23154	n.d.	0	0	0	0	0	1	0	0	0	0
"	EBS 1846	24267, 2 genotypes	6-7	0	0	0	0	0	1	0	0	1/0	1
"	GKLS 7219	23047, 2 genotypes	5-7	0	0	0	0	0	1	0	0	1/0	1
"	UGN 4561	24270	5-6	0	0	0	0	0	1	0	0	0	1
S. microdontum	OCHS 15534	no	n.d.	0	0	0	0	0	0	0	0	0	0
"	HHR 3690	18126	n.d.	0	0	0	0	0	0	0	0	0	0
"	CIP 761088	no	n.d.	0	0	0	0	0	0	0	0	0	0
"	CIP 762314	no	n.d.	0	0	0	0	0	0	0	0	0	0
"	HOHH 6012	25390	5	0	0	0	0	0	1	0	0	0	1
"	n.d.	25385 (613-1)	5	0	0	0	0	0	1	0	0	0	0
"	n.d.	25385 (613-2)	n.d.	0	0	0	0	0	1	0	0	0	1
S. microdontum ssp. gigantophyllum	n.d.	5399	5-9	0	0	0	0	0	1	0	0	1	1
"	n.d.	5414	5	0	0	0	0	0	0	0	0	0	1

″	195185 × 195200	21499	8	0	0	0	0	0	0	0	0	0	0
S. venturii	OKA7584 ×7585	25397 (1)	2.7-8.0	0	1	1	0	1	0	0	0	0	1
″	″	25397 (2)	6	0	0	0	0	0	1	0	0	1	1
″	HOHH 6033	25396	n.d.	0	0	0	0	0	1	0	0	0	1
″	HHR 3741	25398	7-9	0	0	0	0	0	1	0	0	1	1
″	OKA 4388 × 4392	12658	7	0	0	0	0	0	1	0	0	0	1
″	OKA 4388 × 4404	25394	6-7	0	0	0	0	0	1	0	0	0	1
S. sucrense	nd	9753 (D20-30)	n.d.	0	0	0	0	0	0	0	0	0	1
″	nd	9247 (D21-28)	n.d.	0	0	0	0	0	1	0	nd	0	0
S. vernei	OKA 5700	23771 (D20-44)	n.d.	0	1	0	0	0	1	0	0	0	0
″	EBS 181	23767 (D21-973-1)	5	0	0	0	0	0	1	0	0	0	0
″	HAW 3505	18159 (D21-972-2)	n.d.	0	0	0	0	0	1	0	0	0	1
″	OKA 7502	20332	5	0	0	0	0	0	0	0	0	0	0
S. verrucosum	SHGRF 4019	23015	6	0	0	0	0	0	0	0	0	0	0
″	CPC 1340.2	24991	3	0	0	0	0	0	0	0	0	0	0
″	HAW 1658	24995 (CD 410)	7	0	0	0	0	0	0	0	0	0	0
″	″	24995	n.d.	0	0	0	0	0	0	0	0	0	0
″	UGN 1289	23760 (556)	5	0	0	0	0	0	0	0	0	0	0
″	″	24996 (D20-95)	n.d.	0	1	0	0	0	0	0	0	0	0
″	COR 14252	21695	n.d.	0	0	0	0	0	0	0	0	0	0
″	″	24990 (125)	7	0	0	0	0	0	0	0	0	0	0
″	″	24990	n.d.	0	0	0	0	0	0	0	0	0	0
″	GRA 327	24992 (127)	3	0	0	0	0	0	0	0	0	0	0
″	nd	24315	5	0	0	0	0	0	0	0	0	0	0
″	TRN 91 F	24313 (CD 401)	5	0	0	0	0	0	0	0	0	0	0

Table S6 Screening *Solanum* species *Tuberosa* (cultivated species) series with SCAR markers of the *Rpi* genes and for late blight resistance

Species	Plant ID	Accessions No in VIR catalogue	LB resistance	***Rpi-R1***	***Rpi-R2/Rpi-blb3***		***Rpi-R3a***	***Rpi-R3b***	***Rpi-R8***	***Rpi-blb1/Rpi-sto1***		***Rpi-blb2***	***Rpi-vnt1***
				SCAR markers of the *Rpi* genes									
				Rpi-R1-1205	Rpi-R2-1137	Rpi-blb3-305	Rpi-R3a-1380	Rpi-R3b-378	Rpi-R8-1258	Rpi-blb1-821	Rpi-sto1-890	Rpi-blb2- 976	Rpi-vnt1-612
S. phureja	CIP 705164	n.d.	n.d.	0	0	0	0	0	0	0	0	0	1
"	DOP 130 x 131	n.d.	n.d.	0	0	0	0	1	1	0	0	0	1
"	OCH 5156	16533	n.d.	0	0	0	0	1	0	0	0	0	1
"	nd	6502	n.d.	0	0	0	0	0	1	0	0	0	0
"	nd	8940	1.0-4.4	0	0	0	0	0	1	0	0	0	0
"	nd	17618	n.d.	0	0	0	0	0	1	0	0	0	1
S. stenotomum	nd	9278	6.6	0	0	0	0	0	1	0	0	0	1
"	nd	17486	6.2	0	0	0	0	0	0	0	0	0	0

Table S7 Screening *Solanum maglia* (*Maglia* series) with SCAR markers of the *Rpi* genes and for late blight resistance

Plant ID	Accessions No in VIR catalogue	LB resistance	***Rpi-R1***	***Rpi-R2/Rpi-blb3***		***Rpi-R3a***	***Rpi-R3b***	***Rpi-R8***	***Rpi-blb1/Rpi-sto1***		***Rpi-blb2***	***Rpi-vnt1***
			SCAR markers of the *Rpi* genes									
			Rpi-R1-1205	Rpi-R2-1137	Rpi-blb3-305	Rpi-R3a-1380	Rpi-R3b-378	Rpi-R8-1258	Rpi-blb1-821	Rpi-sto1-890	Rpi-blb2-976	Rpi-vnt1-612
nd	24601	n.d.	0	0	0	0	0	1	0	0	0	0
Cor C.1	24604	n.d.	0	0	0	0	0	1	0	0	0	0
CPC 2057	2883	n.d.	0	0	0	0	0	1	0	0	0	0

Table S8. Screening *S. demissum* (*Demissa* series) with SCAR markers of the *Rpi* genes and for late blight resistance

Plant ID	Accessions No in VIR catalogue	LB resistance	***Rpi-R1***	***Rpi-R2/Rpi-blb3***		***Rpi-R3a***	***Rpi-R3b***	***Rpi-R8***	***Rpi-blb1/Rpi-sto1***		***Rpi-blb2***	***Rpi-vnt1***
			SCAR markers of the *Rpi* genes									
			Rpi-R1-1205	Rpi-R2-1137	Rpi-blb3-305	Rpi-R3a-1380	Rpi-R3b-378	Rpi-R8-1258	Rpi-blb1-821	Rpi-sto1-890	Rpi-blb2-976	Rpi-vnt1-612
COR 14283	23306	n.d.	0	0	1	0	1	0	0	0	0	0
COR 14266	21365	n.d.	1	1	0	0	1	0	0	0	0	0
HAW 1296	15174	4-5	1/0	0	0	1/0	0	0	0	0	0	0
HAW 1601	15175	5	1	1	0	0	1	0	0	0	0	0
OCH 14217	18521	8	0	0	0	0	1	0	0	0	0	0
TRHRG 123	19997	n.d	0/1	1	1	0	1	1	0	0	0	1

Table S 9. Screening *Solanum* species *Longipedicellata* series with SCAR markers of the *Rpi* genes and for late blight resistance

Species	Plant ID	Accessions No in VIR catalogue	LN resistance	***Rpi-R1***	***Rpi-R2/Rpi-blb3***		***Rpi-R3a***	***Rpi-R3b***	***Rpi-R8***	***Rpi-blb1/Rpi-sto1***		***Rpi-blb2***	***Rpi-vnt1***
				SCAR markers of the *Rpi* genes									
				Rpi-R1-1205	Rpi-R2-1137	Rpi-blb3-305	Rpi-R3a-1380	Rpi-R3b-378	Rpi-R8-1258	Rpi-blb1-821	Rpi-sto1-890	Rpi-blb2-976	Rpi-vnt1-612
S. stoloniferum	CPC 9	23652	3	0	1	1	1	0/1	0	0	0	0	1
“	GRA 222	24972	3	0	1	0	0	1	n.d.	0	0	1	n.d.
“	GRA 341	24973	3-5	1	0	0	0	0	0	0	0	0	1
"	GRA 366	24420	3-4	0	1	0	0	1	1	0	0	0	1
“	S405	3360	8	0	1	1	0	1	1	0	0	0	1
“	nd	3554	4.0 - 8.4	0/1	1	1	0/1	0	0	0	0	0	1
"	COR 14270	21616	5	0	1	1	0	0	1	0	0	0	1
“	HAW 1520	24976	7-9	0	1	0/1	0	1	1	1	1	0	1
“	CB 82-502	21618	8	0	1	1	0/1	1	0	0	0	0	1
"	TRN 187	24981	8	1	1	1	0	1	0	1	1	0	1
“	TRHRG 21	20106	3	0	0	0	1	1	0	0	0	0/1	1
“	TRHRG 86	nd	n.d.	1	0	0	0	1	1	0	0	0	1
"	nd	24263	4	0	1	1	0	1	1	1	1	0	1
S. polytrichon	OCH 14178	23563	5	1	1	0	0	1	0	0	0	1	1
"	nd	24462	4	0	1	0	0	0	1	0	0	0	1
"	“	24463	4-6	0	1	0	1	0	0	0/1	0	0	0

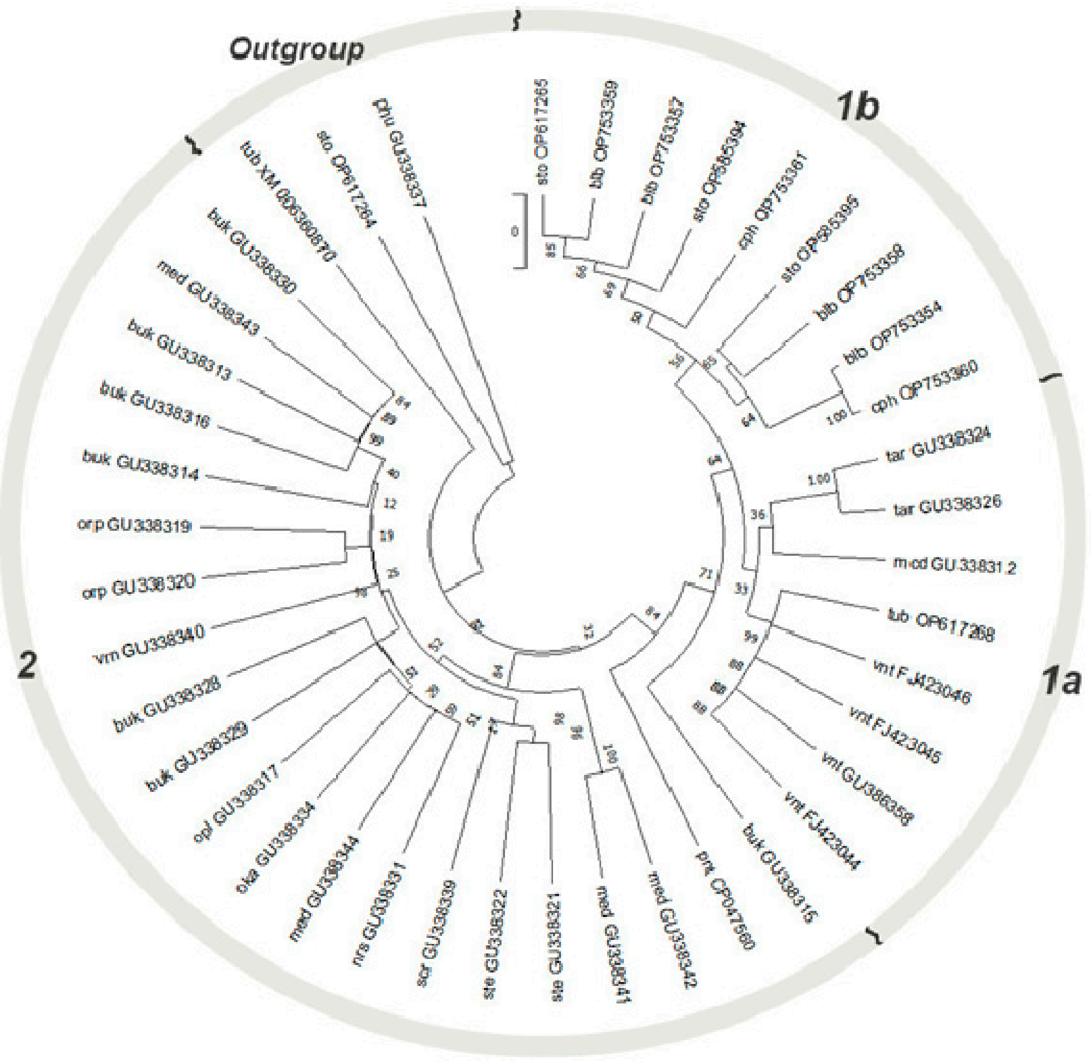

Figure S1. Phylogenetic analysis of the nucleotide sequences of the *Rpi-vnt1* gene and its structural homologues in *Solanum* species section *Petota*.

The species are designated by three-letter abbreviations [68] and the sequences, with the NCBI Genbank accession numbers. The sequences obtained in this study are highlighted in bold. The analysis was performed with the MEGA 10.2.1 program [113] using the Neighbor Joining algorhithm and the bootstrap value of 1000.
